# Prevalence and correlates of current tobacco use and non-user susceptibility to using tobacco products among school-going adolescents in 22 African countries: a secondary analysis of the 2013-2018 global youth tobacco surveys

**DOI:** 10.1186/s13690-022-00881-8

**Published:** 2022-04-14

**Authors:** Peter Bai James, Abdulai Jawo Bah, John Alimamy Kabba, Said Abasse Kassim, Philip Ayizem Dalinjong

**Affiliations:** 1 National Centre for Naturopathic Medicine, Faculty of Health, Southern Cross University, Lismore, New South Wales 2480 Australia; 2 Faculty of Pharmaceutical Sciences, College of Medicine and Allied Health Sciences, University of Sierra Leone, Freetown, Sierra Leone; 3 Institute for Global Health and Development, Queen Margaret University Edinburg, Musselburgh, Scotland, UK; 4 Department of Pharmacy Administration and Clinical Pharmacy, School of Pharmacy, Xi’an Jiaotong University, #76 Yanta West Road, Xi’an, 710061 China; 5 Département de Management, Centre de Recherche en Gestion des Services de Sante, Faculté des sciences de l’administration (FSA), Université Laval (UL), Centre Hospitalière Universitaire (CHU) de Québec, Québec, CA G1V 0A6 Canada; 6 Navrongo Health Research Centre, Upper East Region, Navrongo, Ghana

**Keywords:** Tobacco, Cigarette, Susceptibility, Adolescents, Africa, Global youth tobacco survey

## Abstract

**Background:**

Our study examined the prevalence and associated factors of tobacco product use and non-users’ susceptibility to using tobacco products among school-going adolescents in 22 African countries.

**Methods:**

We analyzed the cross-sectional 2013-2018 Global Youth Tobacco Survey (GYTS) data from 22 African countries. We conducted complex sampling descriptive and logistic regression analyses.

**Results:**

The overall prevalence of current use of any tobacco product among adolescents was 19.1%, with more males (23.7%) than females (13.7%) being current users. Zimbabwe and Morocco were the highest (47.1%) and least (12.6%) reported prevalence, respectively. Being male (AOR = 1.93;95%CI:1.61-2.32), being 16 and older(AOR = 1.37;95%CI:1.01-1.86), exposure to secondhand smoke within (AOR = 1.98;95%CI:1.69-2.32) and outside (AOR = 1.37;95%CI:1.13-1.65) the home, not knowledgeable about the harmful effect of secondhand smoke (AOR = 1.44;95%CI:1.20-1.74), exposure to tobacco industry promotion (AOR = 3.05;95%CI:2.68-3.47) and not in favour of banning smoking in enclosed places (AOR = 1.32;95%CI:1.08-1.60) were associated with current use of any tobacco product.

The prevalence of the susceptibility to using tobacco products among never users of tobacco products was 12.2%, with no significant gender difference. Mozambique (24.6%) and Algeria (4.5%) had the highest and least prevalence of susceptibility to using tobacco products among never users, respectively. Exposure to tobacco industry promotion (AOR = 1.54;95%CI:1.31-1.82), those not in favour of banning smoking in enclosed places (AOR = 1.29;95%CI:1.14-1.45) and those not exposed to anti-smoking school education (AOR = 1.24;95%CI:1.06-1.46) were associated with susceptibility to using any tobacco product among never users of tobacco products.

**Conclusion:**

Our study reports that tobacco use and non-user susceptibility to using tobacco products among school-going adolescents in the 22 African countries is high. As part of public health efforts, governments and other stakeholders need to fully implement anti-tobacco use campaigns, enforce a complete ban on tobacco promotion and advertising, institute educational programs for families, and anti-tobacco use education for the general public and in schools in line with WHO Framework Convention on Tobacco Control guidelines.

**Supplementary Information:**

The online version contains supplementary material available at 10.1186/s13690-022-00881-8.

## Background

Globally, tobacco use is considered a preventable risk factor for morbidity and mortality due to several non-communicable diseases. Tobacco use is considered a global public health threat due to the fact that it has led to more than 200 million deaths and an economic cost of over US$1 trillion in the past three decades [1, 2]. More than 8 million people die each year due to tobacco use, and more than three-fourths of those deaths are due to direct tobacco use [1]. Low- and middle-income countries currently bear the brunt of the current tobacco epidemic as it is host to more than 80% of the world’s tobacco users [3]. By 2030, tobacco-attributable deaths are projected to double in low- and middle-income countries (LMICs), including countries in Africa [3].

Although Africa, compared to other regions of the world, is less impacted by the current tobacco epidemic, it is expected to experience a significant growth in tobacco consumption [4]. Such projected high use of tobacco products is attributed to the urbanization, westernization and change in demographics, as well as increasing penetration of the tobacco industry through its aggressive marketing campaigns targeting primarily young people [5–7]. Although many African countries have signed and ratified the WHO Framework Convention on Tobacco Control (WHO-FCTC), many have not fully implemented robust tobacco control policies as enshrined in the WHO-FCTC guidelines [8]. This has been attributed to governments focussing on more immediate threats due to communicable diseases, the sustained attempts by the tobacco industry to undermine the enactments and implementation of robust laws, corruption, and resistance from the public in some countries as tobacco is considered their source of livelihood [9–13].

Adolescents are known to indulge in high-risk behaviour such as tobacco use. Tobacco use among adolescents is worrisome given the high risk of addiction leading to being a lifetime smoker. A previous African study has shown that adult smokers are two times more likely to have initiated smoking at age 13 and below than those who started at 17 years [14]. The recent global prevalence of tobacco use among school-going adolescents is 17·9% in boys and 11·5% in girls [15]. In Africa, previous studies have reported a slight decline in tobacco use among adolescents from 16·8% in 2006 to 12·3% in 2016 [16, 17]. Despite such decline, strong tobacco control policies are required to prevent adolescents from becoming established smokers.

In addition to implementing robust tobacco control policies, it is crucial for preventive policies and interventions to target non-users of tobacco that are susceptible to using tobacco. Studies have explored susceptibility to using tobacco products, especially cigarettes, globally and outside Africa [18–21]. Globally, one in eight adolescent non-users were susceptible to smoking cigarettes, and a similar prevalence was reported for the African region [20]. The same study also found that exposure to secondhand smoke inside or outside the home, parental or peer smoking, and tobacco industry promotion were risk factors to increased smoking susceptibility [20]. In Africa, an analysis of the 2006-2011 Global Youth Tobacco Survey (GYTS) data from 29 African countries reported that boys compared to girls were more susceptible to cigarette smoking, and that susceptibility to start cigarette smoking was higher among those exposed to secondhand smoking at home than in public places [22].

Although previous global studies have reported the prevalence of tobacco use and susceptibility to using tobacco products in the African region, they have not fully explored the factors that influence tobacco use behaviour in the African region [15, 17, 20]. Also, previous studies that utilized GYTS data from African countries mainly focused on secondhand smoke exposure and susceptibility to initiating cigarette smoking [22–24]. Even though cigarette is the major tobacco product that most studies in Africa have to examine, other non-cigarette tobacco products are known to cause adverse health outcomes. Therefore, a comprehensive evaluation of tobacco use behaviour in the African setting is worth exploring. To our knowledge, no study has comprehensively examined the prevalence and associated factors of tobacco product use and non-users susceptibility to using tobacco products among school-going adolescents in the African region using recent GYTS data. To fill this nascent gap, our study used the most recent GYTS (2013-2018) data from 22 African countries to examine the prevalence and associated factors of tobacco product use and non-users susceptibility to using tobacco products among school-going adolescents in Africa.

## Methods

### Study design and data source

The Global Youth Tobacco Survey (GYTS) 2013-2018 dataset for adolescents aged 11-17 years from 22 African countries were used to conduct our study. The countries included in this study are Algeria, Cameroon, Comoros, Djibouti, Egypt, Gabon, Gambia, Ghana, Kenya, Madagascar, Mauritania, Mauritius, Morocco, Mozambique, Senegal, Seychelles, Sierra Leone, Tanzania, Togo, Tunisia, Uganda and Zimbabwe. The GYTS datasets of these countries are freely available at the WHO Non-Communicable Disease (NCD) microdata repository [25]. GYTS is a questionnaire-based cross-sectional survey conducted among school-going adolescents that uses a two-stage cluster sampling design to examine their tobacco use behaviour. A cluster of schools proportional to enrolment size was chosen, followed by the random selection of classes. We used available data from 22 African countries, and the response rate for each country included in our study is shown in Additional file 1. In line with previous studies [20, 26], we used the most recent dataset for countries in which the GYTS have been conducted more than once.

### Measures

Current tobacco use and susceptibility to using any tobacco product among non-users were our main dependent variables. We defined current tobacco use as the use of cigarettes, smoked tobacco products other than cigarettes and smokeless tobacco products in the last 30 days. Current use of cigarettes was considered as the respondents’ response of “≥ one day to the question “During the past 30 days, on how many days did you smoke cigarettes?“ Also, current use of smoked tobacco products other than cigarettes was defined based on participants self-reported response of “yes“ to the question “During the past 30 days, did you use any form of smoked tobacco products other than cigarettes (such as cigar, pipe water pipe and shisha)?“ In addition, current use of smokeless tobacco product was defined based on respondents’ response of “yes” to the question “During the past 30 days, did you use any form of smokeless tobacco products (e.g. chewing tobacco, snuff, dip)?”

Susceptibility to using tobacco among never tobacco users was measured based on the algorithm developed by Pierce et al. [27]. We identified never-tobacco users as those who have never used cigarettes, smoked tobacco products other than cigarettes and smokeless tobacco products, and they include those who responded “No” to each of the following questions “Have you ever tried or experimented with cigarette smoking, even one or two puffs?”, “Have you ever tried or experimented with any form of smoked tobacco products other than cigarettes (such as cigar, pipe, shisha or water pipe)?” and “Have you ever tried or experimented with any form of smokeless tobacco products (such as snuff, chewing tobacco)?”. Susceptibility to using tobacco was defined by respondents choosing from the following options “(1) definitely not; (2) probably not; (3) probably yes; and (4) definitely yes, based on the following two questions “If one of your best friends offered you a tobacco product, would you use it?” and “At any time during the next 12 months do you think you will use any form of tobacco?“ As it has been previously described [26], we considered respondents who chose “definitely not“ to the two questions as non-susceptible to using tobacco while the rest were considered susceptible. Susceptible and non-susceptible to using tobacco products were recorded as “1″ and “0″ respectively.

Based on the available literature [20, 28–30], 11 independent variables were developed that could potentially predict current tobacco use and susceptibility to using tobacco among never tobacco users. These independent variables include age, sex, access to disposable income, exposure to secondhand smoke (SHS) at home and outside the home, knowledge about harmful effects of SHS, exposure to tobacco industry promotion, favour banning smoking in enclosed places, exposure to anti-smoking media messages and anti-smoking school education. All variables were dichotomized based on previous studies [16, 20, 26]. Additional file 2 gives a detailed description of the dependent and independent variables and how they were recorded.

### Data analysis

The pooled data were analyzed using SPSS version 27. Categorical variables were presented using weighted percentages. Given the established gender difference regarding tobacco use, separate analyses were done for male and female. We employed logistic regression analysis to examine the correlates of the current tobacco use and susceptibility to using tobacco among never-smokers. In our analysis, the prevalence and correlates of the current use of cigarettes, current smoked tobacco products other than cigarettes and current smokeless tobacco products and current tobacco use, as well as susceptibility to using tobacco among never-smokers, were reported. We used complex sampling analysis on SPSS to account for sample weights, sampling design effect. All associations were considered statistically significant if the *p*-value was less than 0.05 with a 95% confidential interval in our regression analysis. The model fitness specification was done with the Hosmer–Lemeshow test, and all the models for each were found to be fit. Multicollinearity was checked using the variance inflation factor (VIF) and all the inflation factors were below 1.20 (see Additional file 3).

### Ethics consideration

We did not obtain ethics approval for this study, given that the GYTS data are de-identified and publicly available. The GYTS from the 22 African countries received ethics approval from their respective Ministries of Health. Besides, a written informed consent was obtained from the parents and guardians of the students who filled the survey questionnaires.

## Results

Table 1 provides details of the study population. The study population consisted of 90,853 school-going adolescents from 22 African countries. Half of them were males (50.3%), and more than two-thirds were between the ages 13-15 years (68.7%). Close to one in three school-going adolescents had been exposed to secondhand smoke at home (28.3%), and more than half were exposed to secondhand smoke outside their homes (57.9%). Less than one in five were exposed to tobacco industry promotion (13.8%), whereas more than two-thirds favoured banning smoking in enclosed places (69%).

**Table 1:** Sample distribution by country population characteristics of male and female school-going adolescents in 22 African Countries using the 2013-2018 Global Youth Tobacco Surveys

Characteristics	Variables	Total (%)	Male (%)	Female (%)
Age	≤12 years	10.3	7.5	12.9
	13-15 years	68.7	68.2	69.4
	≥16 years	21.0	24.3	17.7
Access to disposable Income	Yes	74.7	75.1	74.3
	No	25.3	24.9	25.7
Exposure to SHS at home	Yes	28.3	28.2	28.2
	No	71.7	71.8	71.8
Exposure to SHS outside home	Yes	57.9	60.0	55.6
	No	42.1	40.0	44.4
Knowledge about harmful effects of SHS	Yes	85.3	84.3	86.5
	No	14.7	15.7	13.5
Exposed to tobacco industry promotion	Yes	13.8	15.9	11.6
	No	86.2	84.1	88.4
Favour banning smoking in enclosed places	Yes	69.0	68.0	70.2
	No	31.0	32.0	29.8
Exposure to antismoking media messages	Yes	61.2	61.5	60.8
	No	38.8	38.5	39.2
Antismoking school education	Yes	55.9	55.8	56.3
	No	44.1	44.2	43.7

*SHS* Secondhand Smoke

Table 2 gives detail of the overall prevalence of current use of any tobacco product, prevalence of current cigarette smoking and non-cigarette tobacco product use (current use smoke tobacco product other than cigarette and Current use of smokeless tobacco) and susceptibility to using any tobacco product among never users among male and female adolescents from the 22 African countries. The overall prevalence of current use of any tobacco product among male and female adolescents from the 22 African countries considered in this analysis was 19.1%, with more males (23.7%) than females (13.7%) being current users of any tobacco product. The highest (47.1%) and lowest (12.6%) prevalence of current any tobacco product users were found in Zimbabwe and Morocco, respectively.

**Table 2:** Prevalence of current cigarette smoking, and non-cigarette tobacco products use as well as susceptibility to using any tobacco product among never users among adolescents in 22 African Countries using the 2013-2018 Global Youth Tobacco Surveys

Survey Countries	Current Smokers of Cigarette			Current use of smoke tobacco product other than cigarette			Current use of smokeless tobacco			Current use of any tobacco product			Susceptibility to using any tobacco product among never users of tobacco product		
	Male %^a^	Female %^a^	Total %^a^	Male %^a^	Female %^a^	Total %^a^	Male %^a^	Female %^a^	Total %^a^	Male %^a^	Female %^a^	Total %^a^	Male %^a^	Female %^a^	Total %^a^
All Countries	15.2	6.5	10.9	6.1	2.5	4.3	5.1	3.2	4.1	23.7	13.7	19.1	11.8	12.7	12.2
Algeria	22.1	2.0	11.7	10.2	1.4	5.6	11.3	1.1	6.0	28.9	5.4	16.8	4.7	4.3	4.5
Cameroon	15.0	7.6	11.6	4.3	2.4	3.4	5.2	2.6	4.0	21.3	12.5	17.2	16.6	21.2	18.8
Comoros	18.5	9.9	14.3	3.9	3.2	3.5	5.3	3.0	4.1	26.6	19.9	23.4	17.2	19.6	18.3
Djibouti	22.8	16.5	20.5	6.4	5.5	6.1	9.7	4.9	7.6	34.1	28.8	32.3	19.7	22.8	20.9
Egypt	19.1	4.2	12.0	10.3	2.5	6.4	4.9	4.5	4.8	29.5	11.8	21.0	10.1	10.3	10.1
Gabon	17.4	7.6	12.8	4.4	3.4	3.9	3.6	2.7	3.2	24.4	14.7	19.8	8.2	13.5	10.9
Gambia	21.1	7.3	14.3	7.2	2.3	4.7	2.6	1.1	1.8	30.6	14.1	22.4	12.2	18.0	15.1
Ghana	12.0	9.3	10.7	4.5	3.7	4.4	3.2	4.0	3.6	20.2	18.0	19.4	15.5	16.8	16.0
Kenya	11.6	6.4	9.2	3.0	2.0	2.6	5.8	3.7	4.8	19.4	12.5	16.2	14.5	17.8	16.0
Madagascar	29.1	11.5	20.3	5.3	1.6	3.5	2.8	1.4	2.1	33.4	16.1	**24.7**	11.8	9.0	10.3
Mauritania	23.6	24.6	24.6	7.5	8.0	7.9	6.6	7.0	6.9	39.4	38.3	39.4	13.7	14.3	13.9
Mauritius	27.4	10.0	18.5	13.5	4.4	8.8	2.6	2.5	2.5	34.9	15.2	24.9	6.5	6.4	6.5
Morocco	8.5	2.6	5.6	3.9	2.7	3.3	4.8	2.1	3.4	16.4	8.7	12.6	5.3	6.6	6.0
Mozambique	10.7	11.8	11.7	3.8	4.2	4.2	4.5	3.9	4.3	19.3	20.8	20.7	25.6	23.8	24.6
Senegal	16.2	6.8	12.3	6.5	2.7	4.8	6.5	2.7	4.8	25.7	15.1	21.5	17.5	20.8	18.9
Seychelles	27.8	15.3	21.6	12.3	7.4	9.8	2.9	0.8	1.9	35.4	22.4	**29.0**	9.8	9.1	9.5
Sierra Leone	13.4	6.1	10.2	7.2	2.9	5.9	6.6	5.1	6.8	27.9	18.6	24.6	18.5	19.2	18.6
Tanzania	8.4	5.9	8.0	3.7	1.5	2.9	3.1	1.8	2.6	16.2	12.9	15.6	5.4	7.3	6.3
Togo	11.3	3.2	8.2	4.1	2.0	3.3	2.7	1.5	2.2	17.3	8.5	13.9	11.2	14.0	12.4
Tunisia	18.7	2.7	10.7	9.0	1.6	5.3	3.4	1.6	2.5	23.9	6.1	15.1	6.6	5.9	6.3
Uganda	10.8	8.8	9.8	4.7	4.7	4.7	7.0	5.6	6.3	23.5	20.9	22.2	20.6	22.3	21.5
Zimbabwe	38.4	34.4	37.9	9.0	4.3	6.7	7.4	7.5	7.3	47.6	43.3	**47.1**	15.8	16.1	15.4

^a^weighted percentage

The overall prevalence of current cigarette smoke was 10.9, 95%CI:(9.8-12.1%). with huge gender disparity [males: 15.2%;95%CI (13.4-17.1%) compared to females 6.5%;95%CI (5.7-7.5%)]. The highest (37.9%) and lowest (5.6%) prevalence of current cigarette smoke was reported in Zimbabwe and Morocco, respectively. Only 4.3% of school-going adolescents from the 22 African countries were current smokers of tobacco products other than cigarettes, and the highest (9.8%) and lowest (2.6%) were found in Seychelles and Kenya, respectively. Also, the fewer school-going adolescents (4.1%) were currently using smokeless tobacco and the highest (7.6%), and lowest (1.8%) prevalence were observed in Djibouti and Gambia, respectively.

The prevalence of the susceptibility to using tobacco products among never users of tobacco products was 12.2%, with no significant differences between males (11.8%) and females (12.7%). The highest and least prevalence of the susceptibility to using tobacco products among never users of tobacco products was observed in Mozambique (24.6%) and Algeria (4.5%), respectively.

Table 3 summarises the correlates of the current use of any tobacco products use, and we found significant gender difference existed with males being more likely to be current users of tobacco products than their female counterparts (AOR = 1.93; 95%CI:1.61-2.32). Exposure to secondhand smoke within (AOR = 1.98;95%CI:1.69-2.32) and outside (AOR = 1.37;95%CI:1.13-1.65) the home, not knowledgeable about the harmful effect of secondhand smoke (AOR = 1.44;95%CI:1.20-1.74), exposure to tobacco industry promotion (AOR = 3.05;95%CI:2.68-3.47) and not in favour of banning smoking in enclosed places (AOR = 1.32;95%CI:1.08-1.60) were found to be determinant of any tobacco product use among school-going adolescents in the 22 African countries considered in this study. Such associations were observed in males and females. Also, adolescents in most countries were more likely than those in Morocco to be current user of any tobacco product except Kenya, Senegal, Tanzania, and Tunisia, in which no significant association were observed. Specifically, compared to Morocco, school going adolescents in Madagascar, Seychelles, Mauritania, Mauritius and Zimbabwe showed higher odds of using any form tobacco.

**Table 3:** Determinants of current use of any tobacco products among school going adolescents in 22 African Countries using the 2013-2018 Global Youth Tobacco Surveys

	Current use of any tobacco Products AOR (95%CI)		
	Total	Male OR (95%CI)	Female OR (95%CI)
**Sex**			
Male	1.93(1.61-2.32)		
Female	1		
**Age**			
≤ 12 years	1	1	1
13-15 years	1.02(0.74-1.41)	0.79(0. .58-1.07)	1.17(0.75-1.83)
≥ 16 years	1.37(1.01-1.86)	1.19(0.87-1.63)	1.35(0.86-2.11)
**Disposable Income**			
Yes	1.02(0.90-1.16)	1.06(0.90-1.25)	0.94(0.77-1.16)
No	1	1	1
**Exposure to SHS at home**			
Yes	1.98(1.69-2.32)	2.43(2.03-2.91)	1.59(1.25-2.02)
No	1	1	1
**Exposure to SHS outside home**			
Yes	1.37(1.13-1.65)	1.33(1.03-1.71)	1.33(1.02-1.74)
No	1	1	1
**Knowledge about harmful effects of SHS**			
Yes	1	1	1
No	1.44(1.20-1.74)	1.45(1.15-1.84)	1.29(1.04-1.62)
**Exposed to tobacco industry promotion**			
Yes	3.05(2.68-3.47)	2.87(2.43-3.38)	3.32(2.68-4.12)
No	1	1	1
**Favour banning smoking in enclosed places**			
Yes	1	1	1
No	1.32(1.08-1.60)	1.43(1.10-1.86)	1.07(0.88-1.30)
**Antismoking media messages**			
Yes	1	1	1
No	1.04(0. .90-1.19)	1.04(0.88-1.23)	1.02(0.80-1.30)
**Antismoking school education**			
Yes	1	1	1
No	1.11(0.94-1.31)	1.06(0.90-1.25)	1.18(0.90-1.56)
**Countries**			
Morocco	1	1	1
Algeria	1.50(1.16-1.94)	2.33(1.67-3.24)	0.60(0.41-0.86)
Cameroon	1.34(0.10-1.81)	1.34(0.90-1.99)	1.37(0.96-1.98)
Comoros	1.59(1.1-2.14)	1.49(1.04-2.14)	1.74(1.17-2.59)
Djibouti	2.19(1.59-3.03)	1.99(1.37-2.90)	2.62(1.70-4.02)
Egypt	1.79(1.17-2.73)	2.46(1.52-4.01)	1.10(0.48-2.55)
Gabon	1.57(1.21-2.04)	1.53(1.08-2.18)	1.60(1.08-2.37)
Gambia	1.68(1.31-2.16)	1.98(1.45-2.71)	1.34(0.96-1.88)
Ghana	1.64(1.2-2.18)	1.40(0.98-2.00)	2.05(1.41-3.00)
Kenya	1.16(0.85-1.58)	1.12(0.74-1.67)	1.29(0.87-1.92)
Madagascar	2.59(1.80-3.72)	2.79(1.89-4.12)	2.15(1.34-3.43)
Mauritania	2.73(1.92-3.89)	2.25(1.41-3.58)	3.46(2.38-5.015)
Mauritius	2.47(1.84-3.32)	3.18(2.19-4.60)	1.71(1.13-2.60)
Mozambique	1.42(1.08-1.88)	1.12(0.80-1.56)	1.97(1.40-2.78)
Senegal	1.37(0.98-1.90)	1.44(0.92-2.26)	1.35(0.91-2.00)
Seychelles	2.53(1.94-3.30)	2.39(1.72-3.33)	2.65(1.88-3.74)
Sierra Leone	1.49(1.05-2.11)	1.43(0.95-2.15)	1.58(1.03-2.40)
Tanzania	1.18(0.91-1.55)	1.04(0.74-1.45)	1.40(1.04-1.90)
Togo	1.18(0.89-1.58)	1.24(0.88-1.76)	1.03(0.72-1.48)
Tunisia	1.10(0.85-1.43)	1.51(1.80-2.11)	0.55(0.37-0.81)
Uganda	2.08(1.58-2.73)	1.72(1.20-2.46)	2.64(1.70-4.09)
Zimbabwe	2.99(2.05-4.36)	2.28(1.41-3.68)	3.96(2.60-6.03)

*SHS* Secondhand Smoke, *AOR* Adjusted Odd Ratios, *OR* Odd Ratios

Table 4 summarises the determinants of current cigarette smoking, smoked tobacco other than cigarette and smokeless tobacco among male and female school-going adolescents in the 22 African countries. Males compared to females were more likely to be current cigarette smokers (AOR = 2.65; 95%CI: 2.22- 3.16). Exposure to secondhand smoke inside (AOR = 2.45; 95%CI:2.03- 2.96) and outside (AOR = 2.27; 95%CI:1.88- 2.73) the home was associated with current cigarette smoking, and such association pattern was observed in males and females. Also, not knowledgeable about the harmful effect of secondhand smoke (AOR = 1.55;95%CI: 1.20-2.01),exposure to tobacco industry promotion AOR = 3.22;95%CI:2.71-3.83), not in favour of banning smoking in enclosed places (1.70(1.29- 2.23) and not exposed anti-smoking media messages (AOR = 1.31;95%CI:1.15- 1.48) were associated with current cigarette smoking among school-going adolescents in the 22 African countries considered in this study. Similar associations were found among males and females.

**Table 4:** Determinants of current use of cigarette, smoked tobacco other than cigarette and smokeless tobacco among school going adolescents in in 22 African Countries using the 2013-2018 Global Youth Tobacco Surveys

	Current use of cigarette AOR (95%CI)			Current use of smoked tobacco other than cigarette AOR (95%CI)			Current use of Smokeless tobacco product AOR (95%CI)		
	Total	Male OR (95%CI)	Female OR (95%CI)	Total	Male OR (95%CI)	Female OR (95%CI)	Total	Male OR (95%CI)	Female OR (95%CI)
**Sex**									
Male	2.65(2.22-3.16)			2.34(1.63-3.35)			1.41(0.98-2.02)		
Female	1			1			1		
**Age**									
≤ 12 years	1	1	1	1	1	1	1	1	1
13-15 years	1.07(0.80-1.42)	0.98(0.71-1.36)	0.98(0.67-1.43)	0.90(0.51-1.57)	0.66(0.34-1.28)	1.02(0.54-1.92)	0.98(0.54-1,79)	0.52(0.28-0.96)	1.96(0.78-4.94)
≥ 16 years	1.54(1.16-2.04)	1.62(1.17-2.27)	1.10(0.73-1.68)	1.31(0.74-2.31)	1.00(0.52-1.91)	1.56(0.78-3.11)	1.58(0.89-2.82)	1.10(0.59-2.06)	1.80(0.72-4.52)
**Disposable Income**									
Yes	1.04(0.84-1.27)	1.03(0.83-1.29)	1.06(0.78-1.45)	1.04(0,77-1.40)	1.16(0.77-1.76)	0.95(0.59-1.54)	0.82(0.61-1.12)	0.76(0.53-1.09)	0.94(0.61-1.47)
No	1	1	1	1	1	1	1	1	1
**Exposure to SHS at home**									
Yes	2.45(2.03-2.96)	2.55(2.05-3.18)	2.60(2.02-3.34)	2.09(1.55-2.80)	2.37(1.66-3.40)	2.08(1.44-2.99)	1.83(1.44-2.33)	2.36(1.94-2.88)	1.19(0.68-2.07)
No	1	1	1	1	1	1	1	1	1
**Exposure to SHS outside home**									
Yes	2.27(1.88-2.73)	2.19(1.69-2.83)	2.08(1.67-2.59)	1.24(0.71-2.18)	1.11(0.50-2.47)	1.44(1.07-1.93)	1.20(0.77-1.87)	1.38(1.07-1.75)	1.07(0.50-2.31)
No	1	1	1	1	1	1	1	1	1
**Knowledge about harmful effects of SHS**									
Yes	1	1	1	1	1	1	1	1	1
No	1.55(1.20-2.01)	1.48(1.07-2.05)	1.46(1.10-1.92)	1.38(1.04-1.82)	1.34(0.98-1.83)	1.18(0.78-1.79)	1.33(0.10-1.79)	1.62(1.17-2.23)	0.96(0.63-1.49)
**Exposed to tobacco industry promotion**									
Yes	3.22(2.71-3.83)	2.65(2.16-3.25)	4.78(3.62-6.32)	4.23(3.39-5.27)	4.05(3.13-5.24)	4.56(3.08-6.78)	4.15(3.27-5.26)	4.31(3.13-5.94	3.89(2.78-5.43)
No	1	1	1	1	1	1	1	1	1
**Favour banning smoking in enclosed places**									
Yes	1	1	1	1	1	1	1	1	1
No	1.70(1.29-2.23)	1.79(1.28-2.52)	1.35(1.08-1.680)	1.17(0.89-1.55)	1.13(0.83-1.56)	1.18(0.81-1.72)	0.96(0.74-1.24)	0.92(0.72-1.18)	0.99(0.59-1.67)
**Antismoking media messages**									
Yes	1	1	1	1	1	1	1	1	1
No	1.31(1.15-1.48)	1.33(1.15-1.53)	1.26(1.01-1.57)	0.87(0.64-1.17)	0.72(0.51-1.02)	1.31(0.97-1.77)	0.90(0.66-1.22)	0.97(0.71-1.33)	0.74(0.41-1.34)
**Antismoking school education**									
Yes	1	1	1	1	1	1	1	1	1
No	1.13(0.97-1.31)	1.14(0.95-1.36)	1.10(0.89-1.35)	0.88(0.66-1.18)	0.87(0.60-1.27)	0.92(0.63-1.36)	0.84(0.60-1.17)	0.78(0.59-1.05)	0.89(0.46-1.73)
**Countries**									
Morocco	1	1	1	1	1	1	1	1	1
**Algeria**	2.76(2.01-3.80)	3.60(2.48-5.21)	0.94(0.57-1.57)	1.68(1.09-2.59)	2.88(1.80-4.60)	0.49(0.27-0.87)	1.70(1.10-2.61)	2.27(1.38-3.74)	0.56(0.28-1.13)
**Cameroon**	2.36(1.53-3.62)	2.15(1.31-3.53)	2.91(1.64-5.16)	0.76(0.46-1.28)	0.88(0.52-1.49)	0.69(0.32-1.48)	0.93(0.58-1.48)	0.79(0.46-1.36)	1.19(0.62-2.29)
**Comoros**	2.35(1.64-3.35)	2.16(1.40-3.32)	2.73(1.68-4.46)	0.70(0.43-1.14)	0.69(0.40-1.19)	0.74(0.39-1.39)	0.73(0.46-1.16)	0.61(0.35-1.05)	1.00(0.478-2.10)
**Djibouti**	3.11(2.18-4.44)	2.62(1.70-4.02)	4.41(2.61-7.46)	0.98(0.58-1.68)	1.03(0.551.93)	1.07(0.52-2.22)	1.37(0.84-2.23)	1.43(0.82-2.51)	1.14(0.54-2.43)
**Egypt**	2.27(1.40-3.68)	3.00(1.74-5.18)	0.92(0.41-2.04)	1.78(0.84-3.79)	3.05(1.29-7.17)	0.58(0.22-1.49)	1.34(0.63-2.85)	0.97(0.55-1.71)	2.27(0.60-8.67)
**Gabon**	2.47(1.82-3.37)	2.26(1.58-3.23)	2.90(1.79-4.69)	0.93(0.55-1,59)	0.90(0.43-1.85)	0.99(0.49-1.99)	0.76(0.45-1.28)	0.51(0.22-1.18)	1.33(0.70-2.55)
**Gambia**	2.28(1.67-3.12)	2.40(1.67-3.46)	2.06(1.34-3.16)	1.06(0,65-1.73)	1.56(0.91-2.67)	0.55(0.30-1.00)	0.40(0.25-0.63)	0.36(0.21-6.00)	0.44(0.22-0.89)
**Ghana**	2.06(1.42-2.97)	1.63(1.11-2.40)	3.18(1.95-5.18)	1.14(0.70-1.85)	1.22(0.72-2.07)	1.13(0.58-2.23)	0.98(0.61-1.58)	0.66(0.38-1.15)	1.60(0.79-3.25)
**Kenya**	1.48(1.03-2.12)	1.28(0.80-2.03)	1.92(1.19-3.11)	0.57(0.35-0.94)	0.67(0.35-1.27)	0.49(0.23-1.05)	1.12(0.72-1.72)	1.01(0.60-1.72)	1.40(0.70-2.81)
**Madagascar**	5.73(3.61-9.10)	5.49(3.42-8.81)	5.81(3.12-10.83)	0.92(0.48-1.77)	1.23(0.65-2.32)	0.54(0.20-1.46)	0.55(0.31-0.99)	0.42(0.20-0.88)	0.71(0.26-1.93)
**Mauritania**	2.98(2.07-4.28)	2.16(1.40-3.33)	4.76(2.95-7.70)	1.63(0.92-2.88)	1.55(0.844-2.84)	1.74(0.89-3.39)	1.28(0.84-1.96)	1.00(0.55-1.79)	1.91(1.00-3.64)
**Mauritius**	4.89(3.35-7.13)	5.31(3.37-8.35)	4.19(2.45-7.15)	2.74(1.64-4.60)	4.15(2.45-7.03)	1.35(0.89-3.39)	0.65(0.42-1.01)	0.47(0.26-0.85)	0.96(0.47-1.97)
**Mozambique**	1.88(1.35-2.62)	1.20(0.82-1.75)	3.75(2.43-5.80)	0.85(0.53-1.38)	0.79(0.46-1.35)	0.98(0.55-1.76)	0.90(0.58-1.37)	0.73(0.44-1.22)	1.31(0.69-2.50)
**Senegal**	1.94(1.23-3.04)	1.95(1.15-3.32)	1.97(1.09-3.54)	0.92(0.49-1.72)	1.17(0.58-2.35)	0.76(0.34-1.74)	0.98(0.51-1.91)	0.92(0.41-2.05)	1.06(0.49-2.29)
**Seychelles**	4.71(3.38-6.55)	3.90(2.67-5.68)	6.31(4.07-9.77)	2.42(1.53-3.80)	2.78(1.70-4.55)	1.95(1.07-3.54)	0.36(0.20-0.63)	0.37(0.19-0.73)	0.25(0.10-0.67)
**Sierra Leone**	1.37(0.94-1.98)	1.29(0.83-2.00)	1.59(0.97-2.63)	0.91(0.52-1.62)	1.30(0.68-2.48)	0.53(0.25-1.13)	0.74(0.38-1.46)	0.55(0.45-1.23)	1.16(0.48-2.76)
**Tanzania**	1.37(0.97-1.92)	1.09(0.72-1.65)	2.03(1.26-3.27)	0.60(0.35-1.04)	0.77(0.39-1.51)	0.43(0.22-0.85)	0.68(0.40-1.13)	0.60(0.30-1.21)	0.80(0.36-1.77)
**Togo**	1.97(1.34-2.91)	2.01(1.30-3.10)	1.62(0.98-2.66)	0.97(0.59-1.59)	1.20(0.71-2.04)	0.76(0.40-1.46)	0.62(0.39-0.99)	0.49(0.29-0.84)	0.81(0.41-1.61)
**Tunisia**	1.90(1.35-2.67)	2.47(1.66-3.67)	0.70(0.40-1.21)	1.24(0.79-1.92)	1.95(1.20-3.16)	0.41(0.20-0.87)	0.61(0.37-1.01)	0.55(0.31-1.00)	0.63(0.30-1.34)
**Uganda**	2.22(1.40-3.51)	1.50(0.90-2.51)	4.11(2.24-7.52)	1.25(0.68-2.32)	1.14(0.55-2.33)	1.40(0.68-2.88)	1.52(0.93-2.49)	1.13(0.59-2.17)	2.45(1.08-5.57)
**Zimbabwe**	4.99(3.11-7.99)	3.26(1.91-5.58)	7.98(4.65-13.68)	1.14(0.62-2.08)	1.80(0.89-3.63)	0.52(0.27-1.00)	1.45(0.77-2.73)	0.88(0.46-1.70)	2.69(1.01-7.16)

*SHS* Secondhand Smoke, *AOR* Adjusted Odd Ratios, *OR* Odd Ratios

Also, Table 4 provides factors associated with the current use of smoke tobacco other than cigarettes. Being male (AOR = 2.34;95%CI;1.63-3.35), exposure to secondhand smoke at home (AOR = 2.09;95%CI:1.55- 2.80), exposure to tobacco industry promotion (AOR = 4.23;95%CI:3.39- 5.27) and lack of knowledge about the harmful effects of secondhand smoke (AOR = 1.38;95%CI:1.04- 1.82) were associated with the current use of smoke tobacco other than cigarettes. Exposure to secondhand smoke outside the home was associated with the current use of smoke tobacco other than cigarettes only among females (AOR = 1.44;95%CI:1.07-1.93). As in Table 4, the highest odds of being a current smoker of smoked tobacco other than cigarettes were found in Seychelles (AOR = 2.42;95%CI:1.53-3.80).

In addition, Table 4 shows the factors associated with smokeless tobacco use among our study population. No significant gender difference was observed regarding the use of smokeless tobacco. Exposure to secondhand smoke at home was associated with of smokeless tobacco use (AOR = 1.83; 95CL:1.44- 2.33), but such association was observed only among males (AOR = 2.36;95%CI:1.94-2.88). Exposure to tobacco industry promotion was found to be associated with the use of smokeless tobacco (AOR = 4.15;95%CI:3.27- 5.26), and such association was found in males and females. In comparison with adolescents in Morocco, school-going adolescents in Algeria were more likely to be current users of smokeless tobacco while those in the Gambia and Togo were less likely to be current users of smokeless tobacco.

Table 5 looks at factors associated with susceptibility to using any tobacco product among never users of tobacco products in our study population. We observed that male and female students exposed to tobacco industry promotion (AOR = 1.54;95%CI:1.31-1.82) do not favour banning smoking in enclosed places (AOR = 1.29;95%CI:1.14-1.45) and were not exposed to anti-smoking education in their schools (AOR = 1,24;95%CI;1.06-1.46) were more likely to be susceptible to using any tobacco product among never users of tobacco products. Also, compared to Morocco, adolescents in most countries except Mauritius and Tanzania were found to be more likely and less likely (only Alegria) to be associated with susceptibility to using any tobacco product among never users of tobacco products.

**Table 5:** Determinants of susceptibility to using any tobacco product among never users of tobacco product in school-going adolescents from 22 African Countries using the 2013-2018 Global Youth Tobacco Surveys

	Susceptibility to using any tobacco product among never users of tobacco product AOR (95%CI)		
	Total	Male OR (95%CI)	Female OR (95%CI)
**Sex**			
Male	0.86(0.74-1.01)		
Female	1		
**Age**			
≤ 12 years	1	1	1
13-15 years	0.95(0.79-1.12)	0.84(0.63-1.12)	1.03(0.76-1.38)
≥ 16 years	0.88(0.71-1.11)	0.82(0.60-1.14)	0.91(0.66-1.26)
**Disposable Income**			
Yes	1.14(0.97-1.35)	1.05(0.89-1.24)	1.22(0.93-1.00)
No	1	1	1
**Exposure to SHS at home**			
Yes	0.94(0.79-1.13)	0.90(0.71-1.14)	1.01(0.78-1.30)
No	1	1	1
**Exposure to SHS outside home**			
Yes	0.96(0.84-1.10)	0.85(0.69-1.04)	1.06(0.90-1.25)
No	1	1	1
**Knowledge about harmful effects of SHS**			
Yes	1	1	1
No	0.63(0.53-0.76)	0.59(0.49-0.72)	0.66(0.50-0.89)
**Exposed to tobacco industry promotion**			
Yes	1.54(1.31-1.82)	1.46(1.16-1.85)	1.62(1.29-2.04)
No	1	1	1
**Favour banning smoking in enclosed places**			
Yes	1	1	1
No	1.29(1.14-1.45)	1.33(1.14-1.54)	1.25(1.05-1.48)
**Exposure to antismoking media messages**			
Yes	1	1	1
No	1.03(0.87-1.21)	1.00(0.86-1.17)	1.06(0.82-1.36)
**Antismoking school education**			
Yes	1	1	1
No	1.24(1.06-1.46)	1.15(0.95-1.39)	1.33(1.05-1.70)
**Countries**			
Morocco	1	1	1
Algeria	0.78(0.62-0.99)	0.95(0.69-1.31)	0.67(0.48-0.92)
Cameroon	4.16(2.99-5.79)	3.92(2.68-5.73)	3.48(2.92-6.87)
Comoros	3.58(2.82-4.56)	3.66(2.77-4.84)	3.55(2.46-5.12)
Djibouti	4.59(3.43-6.15)	4.97(3.40-7.26)	4.33(2.96-6/32)
Egypt	1.94(1.27-2.96)	2.19(1.20-4.00)	1.76(1.01-3.07)
Gabon	2.24(1.77-2.84)	1.80(1.28-2.55)	2.61(1.87-3.66)
Gambia	3.16(2.44-4.09)	2.89(2.12-3.93)	3.38(2.38-4.80)
Ghana	3.25(2.50-4.22)	3.46(2.55-4.68)	3.10(2.16-4.45)
Kenya	3.02(2.27-4.03)	2.89(2.02-4.13)	3.17(2.17-4.63)
Madagascar	2.08(1.52-2.85)	2.75(1.87-4.05)	1.59(1.02-2.49)
Mauritania	2.72(2.04-3.61)	2.89(1.99-4.18)	2.57(1.77-3.74)
Mauritius	1.13(0.86-1.48)	1.33(0.85-2.06)	0.98(0.70-1.37)
Mozambique	5.95(4.74-7.45)	6.92(5.16-9.27)	5.12(3.71-7.06)
Senegal	4.12(2.95-5.75)	4.57(3.09-6.76)	3.82(2.51-5.81)
Seychelles	1.73(1.33-2.25)	1.97(1.39-2.81)	1.53(1.05-2.23)
Sierra Leone	3.97(2.98-5.29)	4.44(3.20-6.16)	3.66(2.47-5.40)
Tanzania	1.02(0.77-1.34)	0.90(0.58-1.39)	1.11(0.79-1.56)
Togo	2.49(1.82-3.40)	2.42(1.68-3.50)	2.64(1.78-3.90)
Tunisia	1.08(0.85-1.39)	1.35(0.95-1.92)	0.89(0.62-1.28)
Uganda	4.68(3.59-6.09)	4.66(3.02-7.19)	4.75(3.49-6.46)
Zimbabwe	3.22(2.35-4.40)	4.02(2.53-6.40)	2.64(1.80-3.88)

*SHS* Secondhand Smoke, *AOR* Adjusted Odd Ratios, *OR* Odd Ratios

## Discussion

Our study examines tobacco products use and susceptibility to using any tobacco product among never users in 22 African countries. Our study provides a comprehensive analysis of school-going adolescents’ tobacco product use behaviour in Africa using the most recent GYTS data (2013-2018) from 22 African countries. Although there is a significant disparity in the prevalence of tobacco product use among countries, there is a relatively high prevalence of tobacco use and susceptibility to using any tobacco product among never users. There was a significant gender difference among current cigarette smokers, non-cigarette tobacco products (except smokeless tobacco users), any tobacco product users but not among those susceptible to using any tobacco product. Students exposed to secondhand smoke within and outside their homes, exposure to tobacco industry promotion were more likely to be current tobacco users (cigarette and non-cigarette tobacco products). Being exposed to tobacco industry promotion and not in favour of banning smoking in enclosed places were associated with susceptibility to using any tobacco product among never users of tobacco.

In line with the global prevalence of current cigarette smoking among adolescents 13–15 years in 143 countries [15] and among 12–15 years in 68 low-income and middle-income countries [17], we found that one in ten school-going adolescents in the 22 African countries considered were current cigarette smokers. Our finding was lower than the prevalence reported in the Western Pacific and higher than the rates reported in Europe, Southeast Asia and the Eastern Mediterranean [17]. Our finding was also higher than what was reported for the African region in the 1999-2005 global tobacco use survey among 13-15 years old school-going adolescents using the GYTS dataset [16]. In addition, the rate of cigarette smoking in both males and females in our study was higher than the prevalence reported for males and females in the 1999-2018 GYTS [15]. The difference in prevalence observed may be attributed to differences in the sources of data and the duration of the study considered. We used the recent GYTS data (2013-2018) from 22 African countries, whereas the study by Xi et al. [17] used Global School Health Survey (GSHS) data from 2006 to 2013. Cultural factors among the countries considered maybe a contributing factor as observed in the differences in the use of any tobacco product between countries in our regression analysis. The prevalence of the current use smoke tobacco product other than a cigarette in our study was lower than the global estimate of 11.2%, whereas the prevalence of the current use of any tobacco product was higher than the global estimate using both GYTS and GSHS datasets [15, 17]. In the study by Xi et al. in which they used GSHS data, the prevalence of any tobacco products use in the African region was lower than what we found in our study and this was the case for the Americas, Europe, Southeast Asia, Eastern Mediterranean except for Western Pacific region in which the prevalence was found to be 17.6%. The prevalence of the current use of any tobacco product in our study was also consistent with what was reported for the African region in the 1999-2005 global youth tobacco survey [16]. The prevalence of the current use of any tobacco product for males and females in our study was higher than the one reported for males and females in the 1999-2018 GYTS [15]. Our results have demonstrated that tobacco products use among school-going adolescents in most African countries remains prevalent, and it is highest in Zimbabwe and lowest in Morocco. Such disparity may be attributed to easy access to tobacco products and the weak implementation of anti-tobacco laws in these countries as well as differences in culture. For instance, although Zimbabwe has recently ratified the WHO-FCTC, it remains the largest tobacco producer in Africa and it is known to contribute largely to the country’s economy accounting for 10% of its GDP [12, 31, 32]. It is believed that Zimbabwe’s signing of the WHO-FCTC treaty is undermining efforts to enforce the stipulated measures as few of the laws implemented so far are aimed at reducing tobacco supply or protecting the environment [12]. On the other hand, like other African countries, Morocco has signed and ratified the WHO-FCTC and have laws prohibiting smoking in public places [8]. Even though most countries in the African region have ratified the WHO- FCTC [8], the high prevalence of cigarette smoking and any tobacco product use in our study suggest that little progress has been made over the years in preventing tobacco use among tobacco adolescents in many African countries. The stall in progress may be linked to a lack of human and financial resources to implement tobacco control activities effectively. Also, the increased influence of the tobacco industry and limited public support to implement tobacco control initiatives may explain the slow progress in implementing WHO-FCTC articles.

Regression analysis indicates that exposure to secondhand smoke within and outside the home is associated with tobacco use (cigarette, non-cigarette tobacco products) among school-going adolescents in the 22 African countries. Our finding is in line with previous global surveys in which exposure to secondhand smoke was found to be associated with tobacco use among school-going adolescents globally and in the African region [17, 33] and studies conducted in Asian countries [34, 35], USA [36, 37] and Europe [38]. Our finding suggests that tobacco product use by a family member and smoking in public places, especially enclosed places, is a strong influence on school-going adolescents tobacco use behaviour. Studies have reported that parental use of tobacco products predicts tobacco use among youths [39]. Family and community influence on adolescent tobacco product use may be explained because adolescents are likely to practice high-risk behaviours practised by their parents, siblings, and community members [40]. Even more worrisome is the impact of secondhand smoke on those who are never smokers. A previous study in Africa found that a considerable number of never-smoking adolescents in Africa were exposed to secondhand smoke [41]. In the same study, parental and peer smoking and less exposure anti-smoking media messages were associated with exposure to secondhand smoke [41]. Children’s exposure to secondhand smoke is considered a public health concern as it makes them become potential adult smokers in the future [42] and affects their health, given the hazardous chemicals, they inhale [42, 43]. A recent study among school-going Korean adolescents found that high concentrations of 4-(methylnitrosamino)-1-(3-pyridyl)-1-butanol (NNAL) and cotinine were present in the blood of children of smoking parent compared to those with non-smoking parents [44]. Given the high prevalence of secondhand smoke and its association with tobacco use in our study and the fact that all of the countries considered in our study have rectified WHO-FCTC suggest that more needs to be done in fully implementing article 8 of the WHO-FCTC. This can include implementing educational campaign for family and household members and enforcing a complete smoking ban at home and in public, especially enclosed household spaces.

Similarly, exposure to tobacco industry promotion was associated with tobacco product use (cigarette, non-cigarette tobacco products) among school-going adolescents in the 22 African countries considered in our study. Our findings are consistent with studies conducted elsewhere [45–47]. Our finding may be attributed to the increased market influence of the tobacco industry in many African countries [48]. Such penetration has been attributed to weak legislation on tobacco promotion, less enforcement of existing law, low taxes on tobacco products, and innovative ways by the tobacco industry to reach young people [49–51]. Some of the innovative ways young people have been reached include the use of school programmes and youth camps, provision of scholarships and the use of social media influencers as brand ambassadors [50, 52]. Also, the tobacco industry is known to use subtle ways to influence tobacco control policy development and implementation, and these include acquiring membership of critical policy-making committees and agencies, putting pressure on governments to influence the work of tobacco enforcement institutions as well as the use of their corporate social responsibility programmes to shape public opinion [53]. Also, there have been instances where the tobacco industry actively misled African governments in formulating and implementing tobacco control policies [11]. In addition, a recent report by Jackson and colleagues highlights the unethical dealing of British American Tobacco in Africa to block or weaken tobacco control legislation, and these range from bribery, blackmailing governments to blocking potential competitors [9, 10]. Going forward, governments in Africa need to fully enforce article 13 of the WHO-FCTC framework [54] to ensure that young people are prevented from tobacco industry promotions. The complete ban of all forms of tobacco advertisement, sponsorship and promotion will help to prevent young people from being exposed to a tobacco product. We observed that compared to Morocco, school-going adolescents in Madagascar, Seychelles, Mauritania, Mauritius and Zimbabwe showed higher odds of using any form of tobacco. This finding further aligns with the high prevalence values obtained for these countries. It is also in line with previous studies in which some of these countries have been reported to have high tobacco use [12, 30, 55]. For instance, high use of any tobacco product, especially smokeless tobacco, has been reported in Madagascar. Such high use has been attributed to ethnicity, given that majority of Madagascans have South Asian origin, and this ethnic group are known to be highest users of smokeless tobacco globally [56]. The possible reasons for the higher of tobacco use in Zimbabwe has been explained in the preceding paragraph.

Consistent with previous African studies [29, 30, 57] and recent South Asian countries [47], we did not find any statistically significant association between anti-smoking school education and increased tobacco use. Our finding may be attributed to the absence of or the ineffectiveness of anti-smoking school intervention, which may be explained by the strong influence from friends and family [57, 58]. Current evidence suggests that anti-smoking school interventions have little or no effect on youth smoking behaviour including susceptibility to using tobacco [59, 60]. Currently, data assessing the impact of anti-smoking school interventions on the -school-going adolescent tobacco use are limited in Africa, and this requires further research. In line with recent study in Asia [47],we found in our study that no significant association exist between non-exposure to anti- tobacco media messaging and increase tobacco use. Our finding maybe due to the possibility that current anti- tobacco media messages are not designed to specifically target adolescents in most African countries.

Our study found that less than one in five non-tobacco users were susceptible to using tobacco (12.1%). Our finding is in line with previous global and African estimates [16, 20] and what has been reported in Asian countries [61, 62] but lower among Canadian and U.S. high school students [18, 63] and students in Mexico [19]. With increased penetration of the tobacco industry in Africa, our finding suggests the need for more robust interventions to prevent never-users of tobacco in these countries from initiating tobacco use and eventually becoming tobacco product users in their adulthood. Our finding also highlights the need for governments in these countries to formulate policies and interventions targeting young people in their pre-experimentation stages of tobacco use behaviour. Our study found no gender differences regarding susceptibility to using tobacco among never users as it has been reported in previous studies globally [20], in Africa [22], USA [64] and Pakistan [62] but was consistent with studies conducted in South East Asia countries [61] and in Mexico [19]. Our finding supports the need for governments to adopt gender-neutral policies and interventions when targeting young people susceptible to using tobacco [65]. Consistent with studies conducted globally [20] and in Southeast Asia [55], exposure to tobacco industry promotion was associated with increased susceptibility to using tobacco among non-users. This result further showcases the need for governments to impose a complete ban on tobacco promotion in line with article 13 of the WHO-FCTC framework [54]. Knowledge about the harmful effects of SHS was associated with decreased susceptible to using tobacco among non-users in our study, and our result was in line with studies conducted in other regions [61, 62]. Also, our study shows that those who were against banning smoking in enclosed places were more likely susceptible to using tobacco among non-users, which indicate lack of knowledge about the harmful effects of secondhand smoke. Our findings suggest the need for governments and other stakeholders to provide information to adolescents who are prone to using tobacco products regarding the harmful effects of secondhand smoke. Interventions can include the inclusion of harmful effects of secondhand smoke into high school curriculum, using social media social media influencers to promote the harmful effects of secondhand smoke. Exposure to SHS outside the home was associated with increased susceptibility to smoking among never-smoking males but not females. Our finding is similar to a previous global study although such association was observed in both gender [20]. Such a finding underscores the need for governments to create smoke free public spaces and further strengthen the enforcement of a complete ban on smoking in public enclosed spaces.

In contrast to studies conducted globally [20] and in America [66], exposure to secondhand smoke inside or outside the home and non-exposure to anti-smoking media messaging was not statistically associated with increased susceptibility to using tobacco among non-users of tobacco. Differences in study population and area may explain differences between our studies and others. Even thought our findings did not show statistically significant association, it however reinforces the need to implement smoke free environment at home and in public in African countries. It also points to the need to design anti-smoking media messages that meet the needs of young people.

### Strengths and limitations

To the best of our knowledge, our study is the first to examine the prevalence and correlates of tobacco use and susceptibility to using any tobacco product among school-going adolescents using the most recent GYTS datasets in 22 African countries. Another strength is that nationally representative data from multiple countries were used. Notwithstanding, GYTS is a school-based survey, which means our findings are only representative of school-going adolescents. Also, students provided self-reported responses and were therefore prone to recall bias. In addition, our study did not assess the influence of contextual factors such as the presence or absence of national tobacco control plans across countries as data on such a variable was absent in the datasets. Further, given the cross-sectional nature of the GYTS, we cannot make causal inferences. There is a possibility for disparity in the current rates for some countries, given that we used the most recent data available from the GYTS, which dates back to 2013. However, our analysis is robust enough to be relied on for the studied population.

## Conclusion

Our study has identified that approximately one in five school-going adolescents are current users of any tobacco product in 22 African countries, with the highest and lowest prevalence observed in Zimbabwe and Morocco, respectively. Being male, exposure to SHS in and outside the home, not knowledgeable about the harmful effects of SHS, exposure to tobacco industry promotion and being against banning smoking in enclosed places were associated with the current use of any tobacco product. Less than one in five of never users of tobacco were susceptible to using tobacco in our study. Not being knowledgeable about the harmful effects of SHS, exposure to tobacco industry promotion, and being against banning smoking in enclosed places were significantly associated with susceptibility to using tobacco among never users of tobacco. Our study provides a comprehensive insight into the tobacco use behaviour and an understanding of the early stages of tobacco use behaviour with the aim to implement policies and interventions that stop and prevent tobacco use among adolescents. As part of public health efforts, governments and other stakeholders need to fully implement anti-tobacco use campaigns, enforce a complete ban on tobacco promotion and advertising, institute anti-tobacco use educational programs for families, communities and schools in line with WHO-FCTC guidelines.

## Supplementary Information

**Additional file 1. **Sample distribution by country.

**Additional file 2.** Study measures, survey items with responses, Global Youth Tobacco Survey (GYTS).

**Additional file 3.** Test for multicollinearity

## Data Availability

The datasets analyzed during the current study are publicly available via the WHO NCD Microdata Repository https://extranet.who.int/ncdsmicrodata/index.php/catalog/GYTS

## Abbreviations

CI: Confidential Intervals
FCTC: Framework Convention on Tobacco Control
GYTS: Global Youth Tobacco Survey
OR: Odds ratio
SHS: Second-hand smoke
SPSS: Statistical Package for The Social Sciences
WHO: World Health Organization
