# Supplementary material for: Prevalence and correlates of current tobacco use and non-user susceptibility to using tobacco products among school-going adolescents in 22 African countries: a secondary analysis of the 2013-2018 global youth tobacco surveys
Source: Arch Public Health. 2022 Apr 14;80:121. doi: 10.1186/s13690-022-00881-8 (PMC9009031; doi:10.1186/s13690-022-00881-8)
Supplement: Supplementary file 1 — Additional file 1. Sample distribution by country. [file 13690_2022_881_MOESM1_ESM.docx]

**Additional file 1 Sample distribution by country**

| **Survey Countries** | **Survey Year** | **Percentages** | **Counts** | **Response rate %** |
| --- | --- | --- | --- | --- |
| Algeria | 2013 | 6.9 | 6228 | 96.7 |
| Cameroon | 2014 | 4.5 | 2922 | 88.3 |
| Comoros | 2015 | 0.2 | 2810 | 83.8 |
| Djibouti | 2013 | 0.1 | 1818 | 84.5 |
| Egypt | 2014 | 20.3 | 2471 | 89.2 |
| Gabon | 2014 | 0.6 | 1781 | 85.3 |
| Gambia | 2017 | 0.4 | 12585 | 86.5 |
| Ghana | 2017 | 7.5 | 5664 | 93.8 |
| Kenya | 2013 | 9.0 | 1895 | 90.8 |
| Madagascar | 2018 | 5.4 | 2920 | 96.5 |
| Mauritania | 2018 | 0.7 | 3740 | 91.3 |
| Mauritius | 2016 | 0.2 | 4141 | 89.4 |
| Morocco | 2016 | 7.7 | 3915 | 86.0 |
| Mozambique | 2013 | 3.2 | 5599 | 74.9 |
| Senegal | 2013 | 4.6 | 1728 | 81.3 |
| Seychelles | 2015 | 0.0 | 2485 | 79.8 |
| Sierra Leone | 2017 | 1.2 | 6680 | 79.6 |
| Tanzania | 2016 | 14.3 | 3840 | 92.7 |
| Togo | 2013 | 1.9 | 5298 | 88.0 |
| Tunisia | 2017 | 2.2 | 2448 | 92.8 |
| Uganda | 2018 | 7.8 | 3458 | 75.8 |
| Zimbabwe | 2014 | 1.2 | 6427 | 70.4 |
| Total |  |  | 90853 |  |
