# Supplementary material for: Prevalence and correlates of current tobacco use and non-user susceptibility to using tobacco products among school-going adolescents in 22 African countries: a secondary analysis of the 2013-2018 global youth tobacco surveys
Source: Arch Public Health. 2022 Apr 14;80:121. doi: 10.1186/s13690-022-00881-8 (PMC9009031; doi:10.1186/s13690-022-00881-8)
Supplement: Supplementary file 2 — Additional file 2. Study measures, survey items with responses, Global Youth Tobacco Survey (GYTS). [file 13690_2022_881_MOESM2_ESM.docx]

**Study measures, survey items with responses, Global Youth Tobacco Survey (GYTS)**

| **Study measure** | **GYTS survey items** | **GYTS item responses** | **Dichotomized measure** |
| --- | --- | --- | --- |
| GYTS question used to obtain non-tobacco users’ status | Have you ever tried or experimented with cigarette smoking, even one or two puffs?”, | Yes  No | Yes- Ever smoker  No-Never smoker |
|  | Have you ever tried or experimented with any form of smoked tobacco products other than cigarettes | Yes  No | Yes- Ever smoker  No-Never smoker |
|  | Have you ever tried or experimented with any form of smokeless tobacco products (such as snuff, chewing tobacco)?” | Yes  No | Yes- Ever use smokeless tobacco  No - Never used smokeless tobacco |
| Dependent variable  Tobacco use susceptibility | If one of your best friends offered you a cigarette or other tobacco product, would you use it? | Definitely not  Probably not  Probably yes  Definitely yes | No “Definitely not” for  all three items  Yes any other responses  for any of the three item |
|  | At anytime during the next 12 months do you think you will use any form of tobacco? | Definitely not  Probably not  Probably yes  Definitely yes | No “Definitely not” for  all three items  Yes any other responses  for any of the three item |
| Dependent variable: current tobacco use | During the past 30 days, on how many days did you smoke cigarettes? | 0 days  1 or 2 days  3 to 5 days  6 to 9 days  10 to 19 days  20 to 29 days  All 30 days | No- 0 days  Yes- 1 or more days |
|  | During the past 30 days, did you use any form of smoked tobacco products other than cigarettes (such as cigar, pipe water pipe and shisha)? | Yes  No | Yes  No |
|  | During the past 30 days, did you use any form of smokeless tobacco products (e.g. chewing tobacco, snuff, dip)? | Yes  No | Yes  No |
| Age | How old are you? | 11 years old or younger  12 years old  13 years old  14 years old  15 years old  16 years old  17 years old or older | ≤12years  13-15 years  ≥16 years |
| Sex | What is your sex? | Male  female | Male  female |
| Disposable Income | During an average week, how much money do you have that you can spend on yourself, however you want? | I usually don't have any spending money  Less than “A”  B - C  D - E  F - G  H -I | No Money- I usually don't have any spending money  Have money- any other responses for any of the remaining six items(A-I) |
| SHS exposure in your home | During the past 7 days, on how many days has anyone smoked inside your home, in your presence? | 0 days  1 to 2 days  3 to 4 days  5 to 6 days  7 days | No- 0 days  Yes-1 to 2 days  3 to 4 days  5 to 6 days  7 days |
| SHSb exposure outside home | During the past 7 days, on how many days has anyone smoked in your presence, inside any enclosed public  place, other than your home (such as office, school, shops, restaurants, cinemas, night club)? | 0 days  1 to 2 days  3 to 4 days  5 to 6 days  7 days | No- 0 days  Yes-1 to 2 days  3 to 4 days  5 to 6 days  7 days |
|  | During the past 7 days, on how many days has anyone smoked in your presence, at any outdoor public place  (such as playgrounds, sidewalks, entrances to buildings, parks, beaches, vehicle)? | 0 days  1 to 2 days  3 to 4 days  5 to 6 days  7 days | No- 0 days  Yes-1 to 2 days  3 to 4 days  5 to 6 days  7 days |
| Exposure to antismoking  media messages | During the past 30 days, how many  antismoking media messages (e.g., television,  radio, billboards, posters, newspapers, magazines,  movies) have you seen? | Yes  No | Yes  No |
| Favour banning smoking in enclosed places | Are you in favor of banning smoking inside enclosed public places (such as schools, shops, restaurants, shopping malls, movie theaters)? | No  Yes | No  Yes |
| Knowledge about harmful effects of SHS | Do you think the smoke from other people’s  cigarettes is harmful to you? | Definitely not  Probably not  Probably yes  Definitely yes | No “Definitely not”  Yes - any other responses  for either item |
| Tobacco industry promotion | Do you have something (for example, t-shirt, pen, backpack) with a tobacco product brand logo on it? | No  Yes | No  Yes |
|  | Has a person working for a tobacco company ever offered you a free tobacco product?  . | No  Yes | No  Yes |
| Antismoking school education | During the past 12 months, were you taught in any of your classes about the dangers of tobacco use? | No  I don’t know  Yes | No- No  I don’t know  Yes- Yes |
