# Supplementary material for: Prevalence and correlates of current tobacco use and non-user susceptibility to using tobacco products among school-going adolescents in 22 African countries: a secondary analysis of the 2013-2018 global youth tobacco surveys
Source: Arch Public Health. 2022 Apr 14;80:121. doi: 10.1186/s13690-022-00881-8 (PMC9009031; doi:10.1186/s13690-022-00881-8)
Supplement: Supplementary file 3 — Additional file 3. Test for multicollinearity [file 13690_2022_881_MOESM3_ESM.docx]

**Additional file 3: Test for multicollinearity**

Multicollinearity diagnosis for any tobacco use model

| Independent Variables | Tolerance | VIF |
| --- | --- | --- |
| Sex | 0.991 | 1.009 |
| Age categorised | 0.985 | 1.015 |
| Free money | 0.984 | 1.016 |
| SHS Exposure at Home | 0.879 | 1.138 |
| Knowledge about harmful effects of smoking and SHS | 0.946 | 1.057 |
| Tobacco industry promotion | 0.972 | 1.029 |
| SHS Exposure outside home | 0.854 | 1.171 |
| Favour banning smoking in enclosed places | 0.938 | 1.066 |
| Antismoking media message | 0.948 | 1.055 |
| Antismoking school education | 0.946 | 1.057 |
| Country | 0.966 | 1.035 |

Multicollinearity diagnosis for susceptibility to using tobacco use model

| Independent Variables | Tolerance | VIF |
| --- | --- | --- |
| Sex | 0.991 | 1.009 |
| Age categorised | 0.985 | 1.015 |
| Free money | 0.984 | 1.016 |
| SHS Exposure at Home | 0.879 | 1.138 |
| Knowledge about harmful effects of smoking and SHS | 0.946 | 1.057 |
| Tobacco industry promotion | 0.972 | 1.029 |
| SHS Exposure outside home | 0.854 | 1.171 |
| Favour banning smoking in enclosed places | 0.938 | 1.066 |
| Antismoking media message | 0.948 | 1.055 |
| Antismoking school education | 0.946 | 1.057 |
| Country | 0.966 | 1.035 |
